# Dopamine alleviates cadmium stress in apple trees by recruiting beneficial microorganisms to enhance the physiological resilience revealed by high-throughput sequencing and soil metabolomics

**DOI:** 10.1093/hr/uhad112

**Published:** 2023-05-22

**Authors:** Yang Cao, Peihua Du, Jiran Zhang, Jiahao Ji, Jizhong Xu, Bowen Liang

**Affiliations:** College of Horticulture, Hebei Agricultural University, Baoding, Hebei 071001, China; College of Horticulture, Hebei Agricultural University, Baoding, Hebei 071001, China; College of Horticulture, Hebei Agricultural University, Baoding, Hebei 071001, China; College of Horticulture, Hebei Agricultural University, Baoding, Hebei 071001, China; College of Horticulture, Hebei Agricultural University, Baoding, Hebei 071001, China; College of Horticulture, Hebei Agricultural University, Baoding, Hebei 071001, China

## Abstract

Dopamine has demonstrated promise as a stress-relief substance. However, the function of dopamine in Cd tolerance and its mechanism remains largely unknown. The current study was performed to investigate the mechanism of dopamine on alleviating apple Cd stress through regular application of CdCl_2_ and dopamine solution to potting soil. The results indicated that dopamine significantly reduced reactive oxygen species (ROS) and Cd accumulation and alleviated the inhibitory effect of Cd stress on the growth of apple plants through activation of the antioxidant system, enhancement of photosynthetic capacity, and regulation of gene expression related to Cd absorption and detoxification. The richness of the rhizosphere microbial community increased, and community composition and assembly were affected by dopamine treatment. Network analysis of microbial communities showed that the numbers of nodes and total links increased significantly after dopamine treatment, while the keystone species shifted. Linear discriminant analysis effect size indicated that some biomarkers were significantly enriched after dopamine treatment, suggesting that dopamine induced plants to recruit potentially beneficial microorganisms (*Pseudoxanthomonas*, *Aeromicrobium*, *Bradyrhizobium*, *Frankia*, *Saccharimonadales*, *Novosphingobium*, and *Streptomyces*) to resist Cd stress. The co-occurrence network showed several metabolites that were positively correlated with relative growth rate and negatively correlated with Cd accumulation, suggesting that potentially beneficial microorganisms may be attracted by several metabolites (L-threonic acid, profenamine, juniperic acid and (3β,5ξ,9ξ)-3,6,19-trihydroxyurs-12-en-28-oic acid). Our results demonstrate that dopamine alleviates Cd stress in apple trees by recruiting beneficial microorganisms to enhance the physiological resilience revealed. This study provides an effective means to reduce the harm to agricultural production caused by heavy metals.

## Introduction

Cadmium (Cd) is a heavy metal pollutant with significantly toxicity [1–3]. Moreover, Cd pollution is long-lasting and difficult to remediate [4]. A small amount of Cd can cause abnormalities in plant growth, morphology, and physiology [5]. Cd exposure in plants destroys cell redox homeostasis, which leads to rapid increases in reactive oxygen species (ROS), in turn altering cell membrane function, and allowing the toxicity to spread [6]. Cd stress also causes leaf etiolation, root necrosis, and discoloration, thereby inhibiting key physiological processes such as the absorption and use of water and minerals by plants [5, 7]. The process of Cd absorption and detoxification in plants is regulated by several key genes. It is reported that *MdNRAMP3*, *MdHMA4*, *MdFRO2-like*, *MdHA7* are the key Cd absorption genes, while *MdNAS1* and *MdCAX2* are the key Cd detoxification genes [8, 9]. Cd in the environment is extremely difficult to remove and readily forms organic compounds after absorption by plants [8, 9]. In addition, Cd stress can cause imbalance in the functions of the microbial community [10].

The plant rhizosphere is harboring complex microbial communities [11, 12]. Microbial diversity is a key factor supporting plant growth and ecological stability [13]. However, plant rhizosphere microbial community could be affected by environmental stimuli [14, 15]. Meanwhile, plants seek to cooperate with microbes and recruit beneficial microorganisms from the environment to enhance their capacity to fight adverse conditions [16, 17]. Recruitment of microorganisms by plants under stress conditions is reportedly mediated by root exudates, and these changes tend to enhance plant stress resistance [18]. For example, legumes secrete flavonoids to recruit more nitrogen-fixing bacteria under low-nitrogen conditions [19]; under S-metolachlor stress, wheat attracts potentially beneficial microorganisms with organic acids [20]; and maize enhances its defense responses by releasing benzoxazinoids to attract beneficial microorganisms [21]. Several organic compounds and other molecules such as flavonoids and coumarins have been identified as plant signals that are involved in shaping the rhizosphere microbiota of plants [22]. In addition, rhizosphere microorganisms can directly use low-molecular-weight compounds secreted by roots as carbon sources [23]. Organic compounds secreted by plant roots further shape soil microbial communities and plant root function [24]. Therefore, plant–metabolite–microbe interactions are essential under abiotic stress conditions [25]. However, studies of the mechanisms through which microorganisms affect plant adaptability to stress remain scarce, and the metabolites that regulate microbial communities are yet to be identified.

In recent years, the risk posed by Cd pollution stress in apple orchards has increased sharply, causing a stress response in apples while also seriously impacting fruit yield and quality [26]. Therefore, a method to alleviate Cd stress production is needed. Dopamine is a highly antioxidant amine and the addition of exogenous dopamine to plants has a wide range of benefits [27, 28]. However, elucidating the manner in which dopamine regulates the various processes that confer Cd stress tolerance in plants is also necessary. Some studies have shown potential benefits of dopamine in coordination of beneficial microbes against stress. Dopamine has been widely used in apples to improve resistance to various stresses, including salt [29], drought [30], replant disease [31], waterlogging [28], and nutrient deficiency [32–34]. Moreover, studies have shown that dopamine can alleviate Cd stress by improving photosynthesis, enhancing the activity of antioxidant enzymes and regulating Cd absorption and detoxification genes [8]. However, gaps in current research include the effect of exogenous dopamine on rhizosphere microorganisms, and whether plants can regulate rhizosphere microorganisms through control of root exudates to alleviate Cd stress.

In this study, we hypothesize that exogenous dopamine can affect rhizosphere microbial communities and regulate soil metabolites, and relieve Cd stress by recruiting some potentially beneficial microorganisms and metabolites. To test this hypothesis, *Malus hupehensis* was selected as the model plant. The composition and assembly of microbial communities were analysed and potentially beneficial microorganisms and metabolites were explored by high-throughput sequencing and metabolomics under Cd stress with or without dopamine supplementation. This study provides a new perspective on the stress-relieving properties of dopamine and systematically elucidates the mechanism of dopamine-induced Cd tolerance.

## Results

### Effects of dopamine on apple growth, total chlorophyll content (TCC), relative electrolytic leakage (REL), dopamine content, and photosynthetic parameters under Cd stress

**Figure 1:**
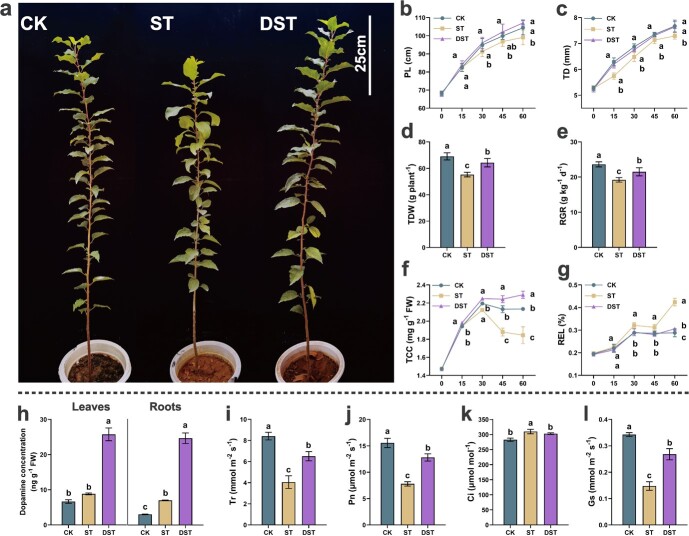
Effects of dopamine on apple growth parameters and dopamine content under Cd stress. (**a** phenotype, (**b**) plant length (PL), (**c**) trunk diameter (TD), (**d**) total dry weight (TDW), (**e**) relative growth rate (RGR), (**f**) TCC, (**g**) REL, (**h**) dopamine content in leaves and roots, (**i**) transpiration rate (Tr), (**j**) net photosynthesis rate (Pn), (**k**) stomatal conductance (Gs), (**l**) intercellular CO_2_ concentration (Ci). Letters indicate significant differences between different treatment (*n* = 5, Tukey’s multiple range tests, *P* < 0.05). CK, control; DST, Cd stress with 100 μM dopamine; ST, Cd stress.

At day 60, compared with CK, plant length (PL) (−5.1%), trunk diameter (TD) (−5.0%), total dry weight (TDW) (−20.0%), and relative growth rate (RGR) (−18.6%) of ST seedlings were significantly reduced. Conversely, dopamine significantly increased the above parameters by 8.1%, 4.8%, 16.2%, and 12.0%, compared with ST (Fig. 1a–e). In addition, TCC was significantly increased by 7.0% after dopamine treatment compared with ST plants. And REL of ST plants increased significantly by 44.8% compared with CK, while DST plants increased only 6.9% (Fig. 1f and g).

We measured the endogenous levels of dopamine in leaves and roots after 60 days of treatment; compared with CK, the content of dopamine in Cd-stressed roots was significantly elevated. After the application of dopamine, the content of dopamine in root and leaf was significantly increased by 252.6% and 191.7% compared with ST (Fig. 1h), demonstrating that exogenous dopamine application increased endogenous dopamine levels. In addition, the transpiration rate (Tr), stomatal conductance (Gs) and net photosynthesis rate (Pn) of DST plants were significantly increased by 60.3%, 80.0%, and 64.3% compared to ST, while intercellular CO_2_ concentration (Ci) was decreased by 2.3% (Fig. 1i–l).

### Dopamine alleviated Cd-induced oxidative stress

**Figure 2:**
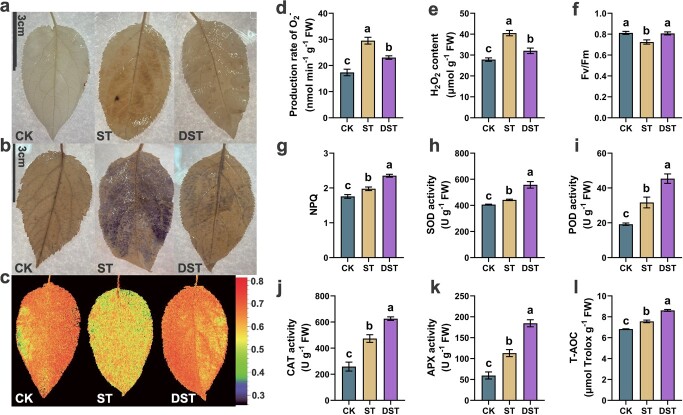
Effects of dopamine on stress-response trait under Cd stress. (**a**, **d**) the levels of superoxide anion (O_2_^•−^) in leaves, (**b**, **e**) the levels of hydrogen peroxide (H_2_O_2_) in leaves, (**c**, **f**) chlorophyll fluorescence parameters (Fv/Fm), (**g**) non-photochemical quenching coefcient (NPQ), (**h**) superoxide dismutase activity (SOD), (**i**) peroxidase activity (POD), (**j**) catalase activity (CAT), (**k**) ascorbate oxidase activity (APX), (**l**) total antioxidant capacity (T-AOC). Letters indicate significant differences in stress-response trait between different treatment (Tukey’s multiple range tests, *P* < 0.05 and *n* = 5). CK, control; DST, Cd stress with 100 μM dopamine; ST, Cd stress.

The H_2_O_2_ content and O_2_^•−^ production rate significantly decreased by 20.9% and 21.9% in DST plants relative to ST. In addition, the chlorophyll fluorescence parameters (Fv/Fm) and non-photochemical quenching coefficient (NPQ) showed significantly increases of 11.4% and 19.0% in DST compared to ST. At the same time, we observed that peroxidase (POD), catalase (CAT), superoxide dismutase (SOD) and ascorbate oxidase (APX) in apple leaves increased significantly in MST plants, with the activity of antioxidant enzymes above significantly increasing by 26.0%, 32.1%, 43.2%, and 63.2%, compared to ST, respectively. Finally, compared to CK, total antioxidation capability (T-AOC) increased significantly by 10.9% under Cd stress and by 26.4% in DST (Fig. 2).

### Dopamine reduced the cd content in apple plant

**Figure 3:**
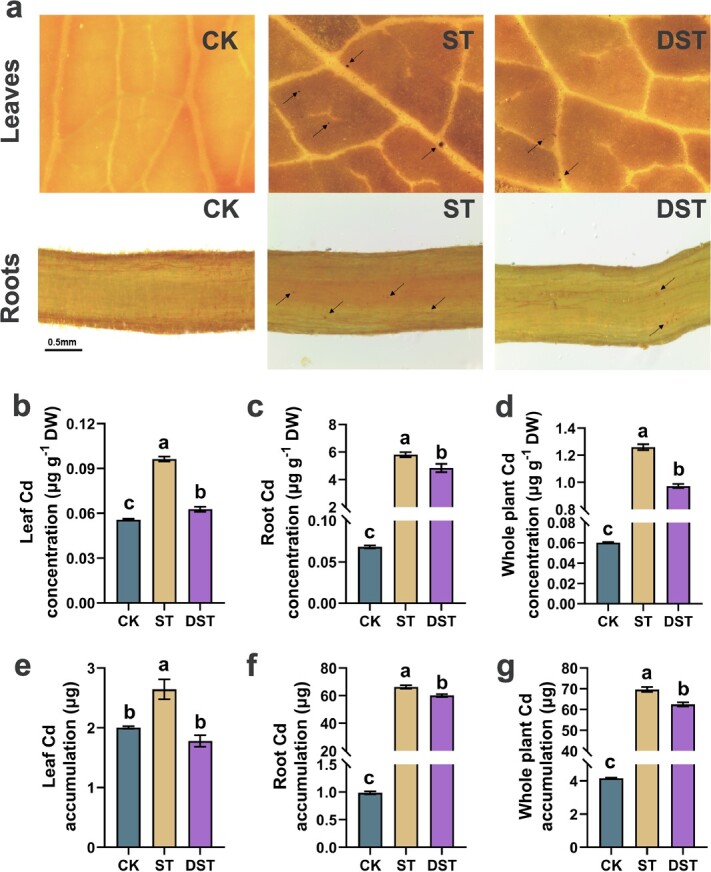
Plant Cd concentration and accumulation. (**a**) Histochemical staining of Cd, (**b**) leaf, (**c**) root, (**d**) whole plant Cd concentration, (**e**) leaf, (**f**) root, (**g**) whole plant Cd accumulation. Letters indicate significant differences in plant Cd content (Tukey’s multiple range tests, *P* < 0.05 and *n* = 5). CK, control; DST, Cd stress with 100 μM dopamine; ST, Cd stress.

Figure 3a shows stained apple leaves and roots after different treatments, where Cd accumulation is visible in the form of a brown-red complex. The Cd concentration and accumulation in ST plants increased significantly by 85.2 and 67.0 times in roots and by 1.72 and 1.32 times in leaves relative to CK plants. However, the Cd concentration and accumulation in DST plants were significantly reduced by 16.8% and 9.3% in roots and by 34.8% and 32.7% in leaves compared to ST plants. The whole-plant Cd concentration and accumulation showed similar trends among treatments (Fig. 3).

### Dopamine regulated antioxidant enzymes and ascorbate (AsA)–Glutathione (GSH) cycle-related genes and Cd uptake and detoxification genes

**Figure 4:**
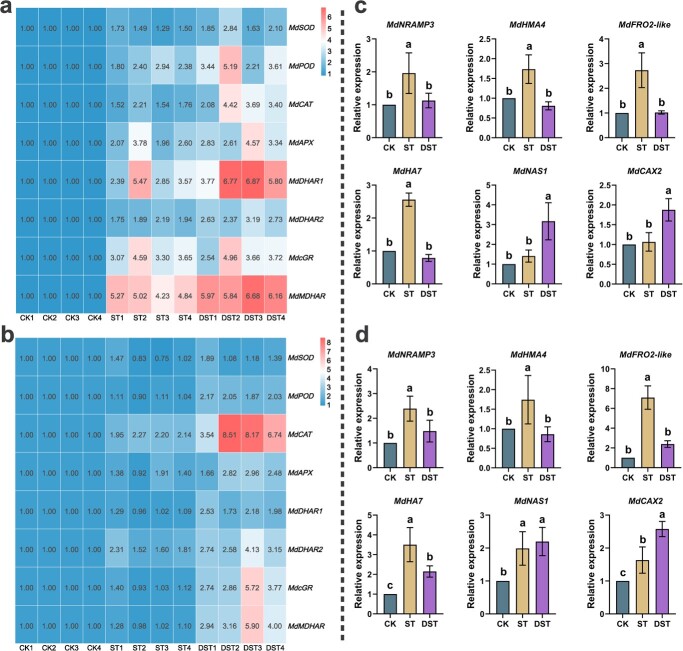
Gene expressions related to antioxidant enzymes and AsA–GSH cycle and Cd uptake and detoxification under different treatment. The heat maps associated with antioxidant enzymes and AsA–GSH cycle related genes in leaves (**a**) and roots (**b**). Plant gene expressions related to Cd absorption and detoxification in leaves (**c**) and roots (**d**). Letters show significant differences in gene expressions (Tukey’s multiple range tests, *P* < 0.05 and *n* = 4). CK, control; DST, Cd stress with 100 μM dopamine; ST, Cd stress.

Most antioxidant enzymes and AsA–GSH cycle-related genes showed varying degrees of up-regulation in ST leaves, and no obvious differences were shown in ST roots. However, almost all antioxidant and AsA–GSH-related genes were markedly up-regulated after dopamine application. Among them, *MdCAT* was up-regulated by 1.9 times in leaves, compared to ST, while *MdCAT*, *MdMDHAR*, and *MdcGR* were up-regulated by 3.2, 3.6, and 3.4 times in roots, respectively (Fig. 4a and b). The expression levels of *MdNRAMP3*, *MdHMA4*, *MdHA7*, and *MdFRO2-like* were down-regulated by 42.3%, 53.2%, 69.1%, and 62.6% in the DST leaves when compared with ST, and by 55.6%, 66.4%, 10.6, and 38.6% in roots, respectively. Among Cd detoxification genes, *MdCAX2* and *MdNAS1* were significantly up-regulated by 76.4% and 125.7% in leaves, and *MdCAX2* was significantly up-regulated by 58.3% in roots (Fig. 4c and d).

### Microbial diversity and community composition

**Figure 5:**
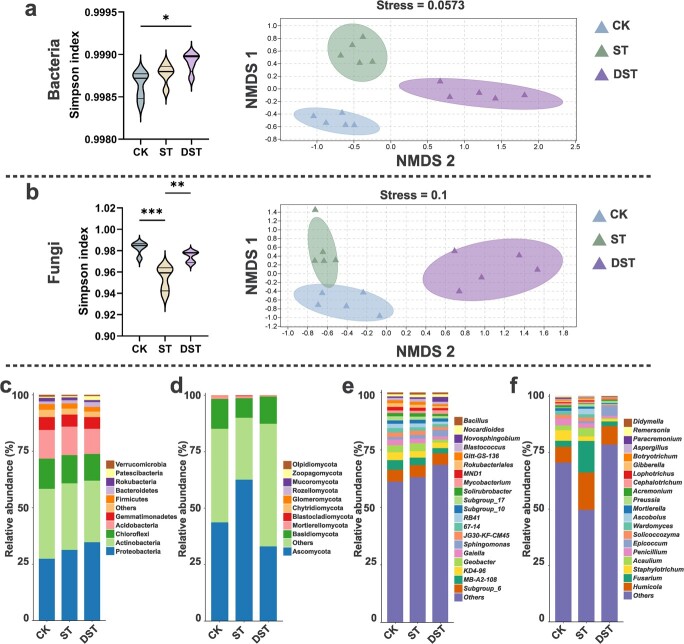
Microbial community composition in rhizosphere soil. (**a**, **b**) Simpson index and NMDS. (**c**, **d**) Relative abundances at the phylum level and (**e**, **f**) at the genus level. (**a**, **c**, **e**) bacteria; (**b**, **d**, **f**) fungi. CK, control; DST, Cd stress with 100 μM dopamine; ST, Cd stress.

Assessment of bacterial and fungal α-diversity after dopamine treatment indicated that the Simpson index increased markedly compared with ST. Nonmetric multidimensional scaling analysis (NMDS) showed that Cd stress and exogenous dopamine treatment resulted in significant differences among treatments (Fig. 5a and b). Acidobacteria, Chloroflexi, Actinobacteria dominated the bacterial communities, while Basidiomycota, Ascomycota dominated the fungal communities in various treatments (Fig. 5c and d). The dominant bacterial order was Betaproteobacteriales, followed by Rhizobiales and Subgroup_6. Sordariales, Hypocreales, and Microascales were the dominant orders in fungal communities (Fig. S1, see online supplementary material). The dominant bacterial and fungal genera also changed, the relative abundances of most dominant bacterial and fungal genera in DST decreased significantly relative to ST (Fig. 5e and f).

### Assembly of the microbial community after various treatments

**Figure 6:**
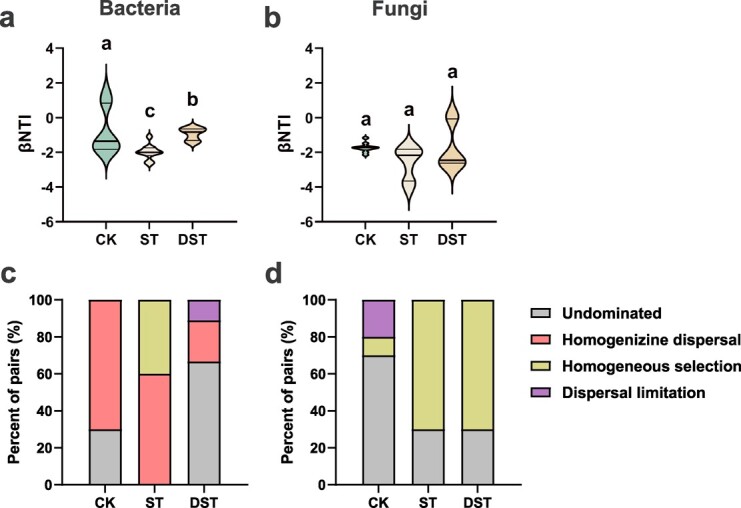
Distribution of β-NTI among bacterial (**a**) and fungal (**b**) samples. Proportion of undominated process, homogenizing dispersal, homogeneous selection and dispersal limitation in the bacterial (**c**) and fungal (**d**) assembly process. CK, control; DST, Cd stress with 100 μM dopamine; ST, Cd stress.

The assembly process (deterministic or stochastic) was evaluated for each treatment. Consistent variable selection was observed in bacterial communities of the CK and DST treatments (|β-nearest taxon index (βNTI) | < 2), while various directions were found within ST samples (|βNTI| > 2). Consistent variable selection was also observed within ST and DST samples of rhizosphere fungal communities (|βNTI| > 2), while various directions were found within CK samples (|βNTI| < 2). In addition, the proportions of various processes affecting microbial assembly were assessed. Homogenizing dispersal (RCbray < −0.95) was dominant among stochastic processes in the rhizosphere bacterial communities of CK and ST. Homogeneous selection was of secondary importance in ST samples. In contrast, no dominant process (i.e., ecological drift), homogenizing dispersal, and dispersal limitation accounted for 66.7%, 22.2%, and 11.1%, respectively, of the microbial assembly in bacterial community data from the DST treatment. For rhizosphere fungal communities, no dominant process, dispersal limitation, and homogeneous selection were the most important ecological processes in CK samples. However, homogeneous selection was dominant in ST and DST samples (Fig. 6).

### Network analysis of microbial communities

**Figure 7:**
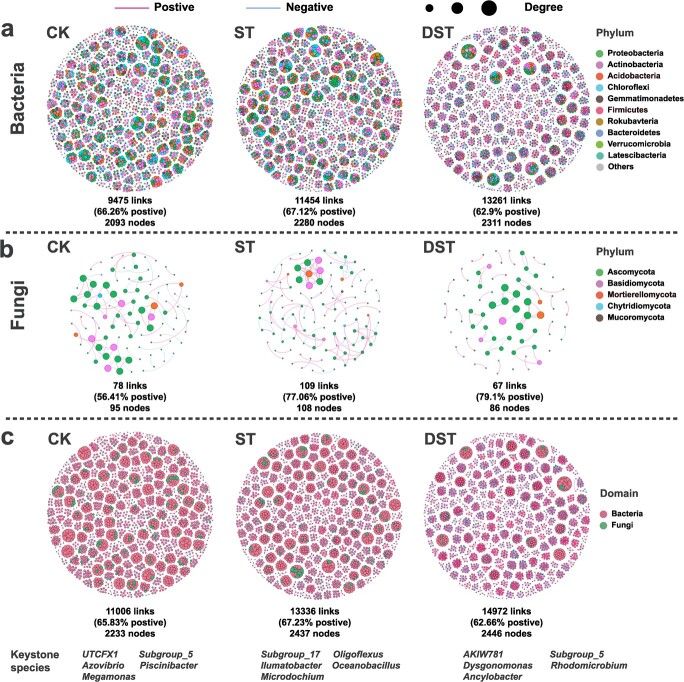
Networks between the bacterial communities (**a**), fungal communities (**b**), bacterial and fungal communities (**c**) in rhizosphere soil. Red lines: positive relationships, and green lines: negative relationships. For each treatment, five keystone species (highest centrality scores) were screened. CK, control; DST, Cd stress with 100 μM dopamine; ST, Cd stress.

Compared to CK, the quantities of nodes and total links between the bacterial and fungal communities increased significantly. In the bacterial community of DST sample, quantities of nodes and total links increased significantly compared to ST. For fungal community, quantities of nodes and total links decreased obviously after dopamine treatment compared to ST, and the quantity of total links between the bacterial and fungal communities increased obviously. In addition, network density was higher for bacteria than for fungi. The top five keystone genera in each treatment were identified based on centrality scores illustrating multiple correlations with other microorganisms. The results indicated that Cd stress and dopamine had significant impacts on microbiome structure (Fig. 7).

### Relationships of the relative abundance of potentially beneficial microorganisms with RGR and whole-plant Cd accumulation

**Figure 8:**
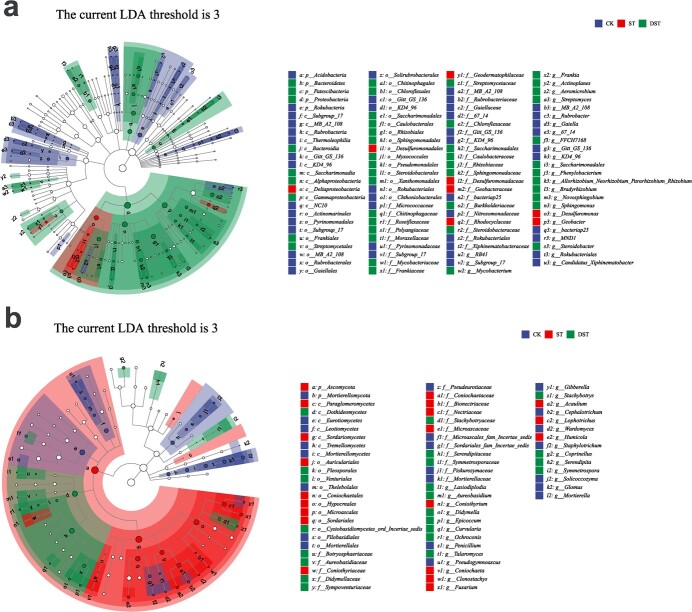
Cladogram showing the phylogenetic distribution of the bacterial (**a**) and fungal (**b**) lineages. CK, control; DST, Cd stress with 100 μM dopamine; ST, Cd stress.

Eighteen groups of bacteria and nine groups of fungi (at the genus level) were significantly enriched in the CK group. Five groups of bacteria and seven groups of fungi were significantly enriched in the ST group: *Geobacter*, *Desulfuromonas*, *TK10*, *Subgroup_7*, *Thauera*, *Fusarium*, *Humicola*, *Acaulium*, *Coniochaeta*, *Lophotrichus*, *Coniothyrium*, and *Clonostachys*. Seventeen groups of bacteria and eleven groups of fungi were significantly enriched in the DST group; the top five bacterial and fungal genera based on LDA score were *Sphingomonas*, *Novosphingobium*, *Actinoplanes*, *Saccharimonadales*, *Steroidobacter*, *Epicoccum*, *Serendipita*, *Didymella*, *Symmetrospora*, and *Coprinellus* (Fig. 8; Figs. S2 and S3, see online supplementary material).

**Figure 9:**
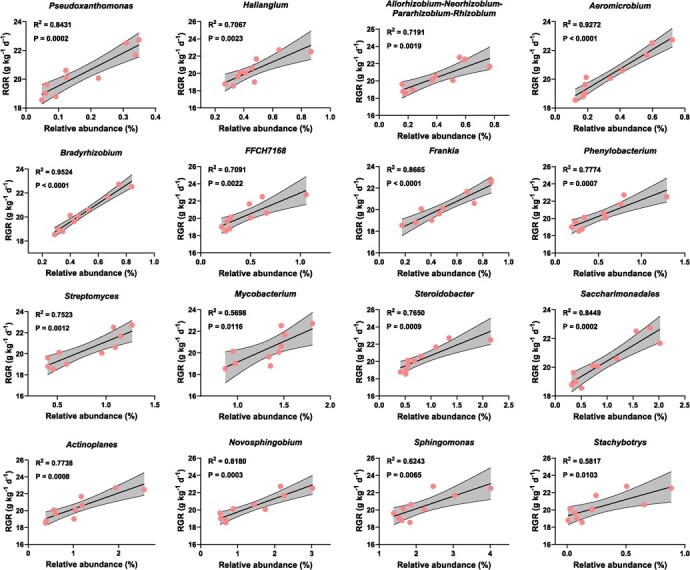
Relationships between the relative abundance of biomarker taxa and relative growth rate (RGR) (Pearson’s correlation analysis). Only the biomarker taxa that are significantly correlated with RGR are shown in the figure.

**Figure 10:**
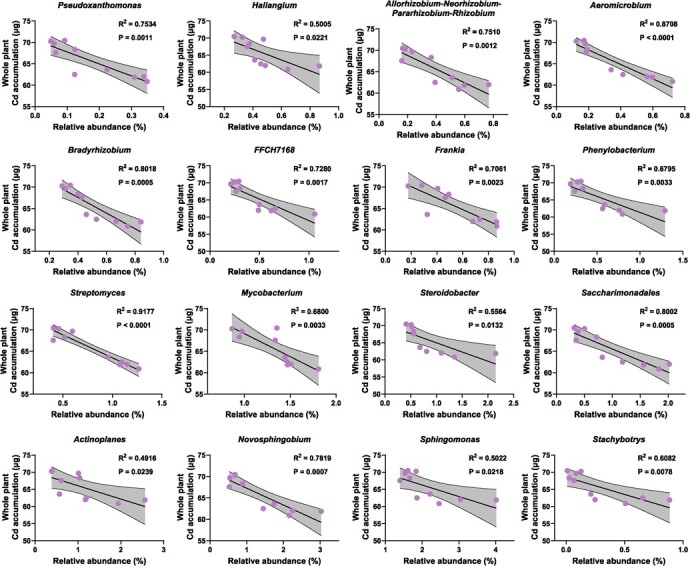
Relationships between the relative abundance of biomarker taxa and whole plant Cd accumulation (Pearson’s correlation analysis). Only the biomarker taxa that are significantly correlated with whole plant Cd accumulation are shown in the figure.

The relative abundances of biomarkers (at the genus level) significantly enriched in the DST group were correlated with RGR and whole-plant Cd accumulation through Pearson correlation analysis (R^2^ > 0.8, *P* < 0.001). The results showed 16 microorganisms (15 bacteria and one fungus) that were significantly correlated with both RGR and whole-plant Cd accumulation. The relative abundances of *Pseudoxanthomonas* (R^2^ = 0.8431), *Aeromicrobium* (R^2^ = 0.9272), *Bradyrhizobium* (R^2^ = 0.9524), *Frankia* (R^2^ = 0.8665), *Saccharimonadales* (R^2^ = 0.8449), and *Novosphingobium* (R^2^ = 0.8180) were strongly positively correlated with RGR (R^2^ > 0.8) (Fig. 9)*.* In addition, the relative abundances of *Aeromicrobium* (R^2^ = 0.8708), *Bradyrhizobium* (R^2^ = 0.8018), *Streptomyces* (R^2^ = 0.9177), and *Saccharimonadales* (R^2^ = 0.8002) were strongly negatively correlated with whole-plant Cd accumulation (Fig. 10).

### Exogenous dopamine modulates soil metabolism in Cd-stressed soil

**Figure 11:**
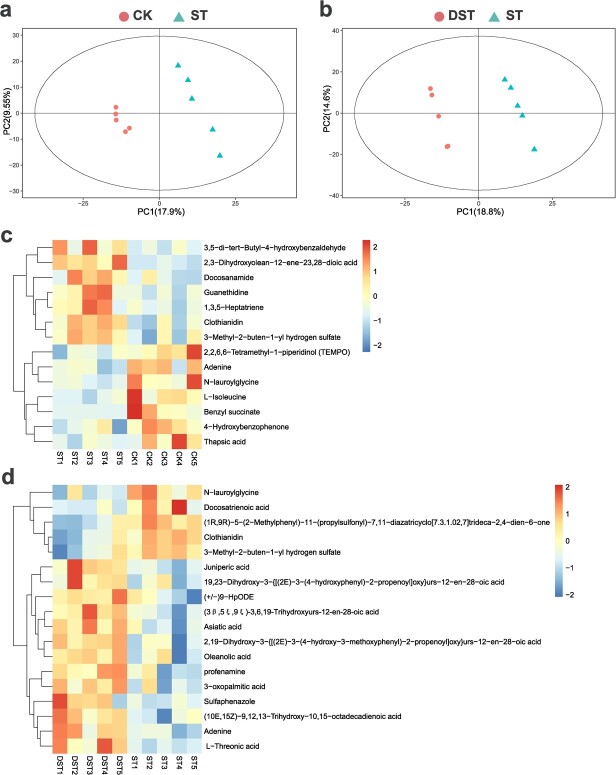
Soil metabolome under different treatments. (**a**, **b**) PLS-DA of soil metabolites. (**c**, **d**) Heatmap showed the relative abundance of differential metabolites among the different treatment groups. CK, control; DST, Cd stress with 100 μM dopamine; ST, Cd stress.

Metabolomics was used to study the soil metabolites in each treatment (VIP > 1, *P* < 0.05). The partial least squares discrimination analysis (PLS-DA) indicated that the treatments were significantly separated. Fourteen and 18 metabolites markedly changed in abundance in response to Cd stress and the addition of dopamine with Cd treatment, respectively. These results indicate that exogenous dopamine obviously altered the soil metabolite profile under Cd stress. After dopamine application, the relative abundances of 13 soil metabolites were obviously up-regulated and those of five soil metabolites were obviously down-regulated compared to ST (Fig. 11).

### Correlations between the rhizosphere microbiome and soil metabolites

**Figure 12:**
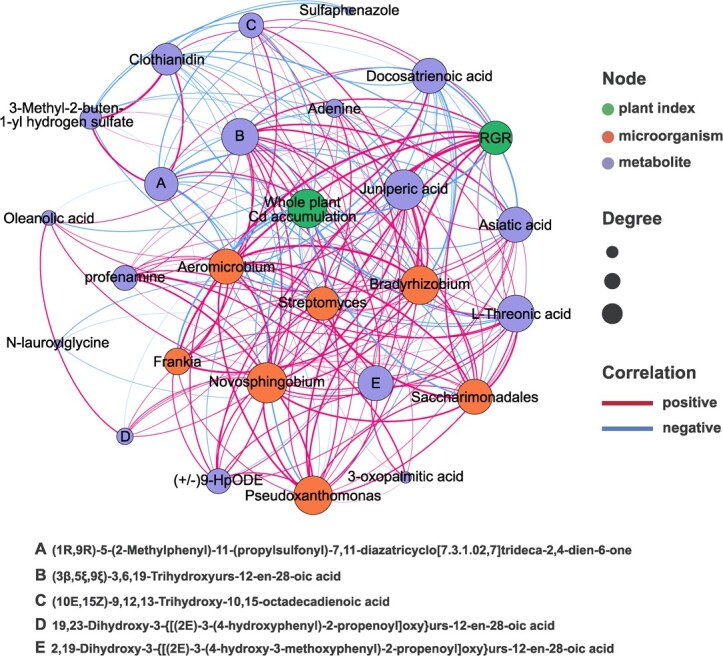
Co-occurrence analysis among biomarker taxa, soil metabolites and relative growth rate (RGR) / whole-plant Cd accumulation. Green dots: RGR / whole-plant Cd accumulation; orange dots: biomarker taxa; purple dots: soil metabolites. CK, control; DST, Cd stress with 100 μM dopamine; ST, Cd stress.

The co-occurrence network was used to illustrate the correlations of potentially beneficial microorganisms with RGR and whole-plant Cd accumulation, as described above, and also demonstrated significant positive correlations between potentially beneficial microorganisms and metabolites. The relative abundances of (3β,5ξ,9ξ)-3,6,19-trihydroxyurs-12-en-28-oic acid (r = 0.8424), (+/−)9-HpODE (r = 0.8424), and juniperic acid (r = 0.7939) were positively correlated with RGR. The relative abundances of (10E,15Z)-9,12,13-trihydroxy-10,15-octadecadienoic acid (r = −0.8182), (3β,5ξ,9ξ)-3,6,19-trihydroxyurs-12-en-28-oic acid (r = −0.8061), and L-threonic acid (r = −0.8061) were negatively correlated with whole-plant Cd accumulation. *Saccharimonadales*, *Pseudoxanthomonas*, and *Novosphingobium* had the strongest positive correlations with L-threonic acid (r = 0.9152), profenamine (r = 0.8788), and juniperic acid (r = 0.8788), respectively. The metabolite (3β,5ξ,9ξ)-3,6,19-trihydroxyurs-12-en-28-oic acid had strong positive correlations with *Aeromicrobium* (r = 0.8545), *Bradyrhizobium* (r = 0.8424), and *Streptomyces* (r = 0.8061) (Fig. 12).

## Discussion

The overuse of metal-based pesticides and fertilizers and the discharge of industrial ‘three wastes’ lead to widespread Cd pollution, and it is difficult for farmers to completely avoid using Cd-contaminated soil [35, 36]. Apple is more susceptible to cadmium stress due to its large planting area, long growth cycle, long time and space span. In addition, cleaning up Cd-contaminated soil is difficult and takes a long time [8, 35]. The tolerance to Cd stress can be improved by applying specific exogenous substances [37], but the mechanism through which dopamine induces Cd stress tolerance has not been systematically investigated. In this study, analysis of growth and physiological indicators, high-throughput sequences, and metabolomic data revealed the effect of dopamine on alleviating Cd toxicity.

Dopamine is an ecologically beneficial option for reducing heavy metal stress [37]. In this study, Cd treatment obviously altered the growth of apple plants, with reductions in PL, TD, TDW, and RGR. However, dopamine obviously alleviated the inhibitory effect of Cd stress (Fig. 1). In addition, numerous common physiological indexes, including TCC, Fv/Fm, and NPQ, as well as measures of the antioxidant enzyme and photosynthesis, were severely impacted by Cd stress (Fig. 2). Dopamine obviously decreased the accumulation of ROS and restored some of the physiological damage caused by Cd stress. Dopamine has physiological benefits for plants [34, 38]. More importantly, the expression levels of antioxidant enzymes (*MdSOD*, *MdPOD*, and *MdCAT*) and ASA-GSH cycling-related genes (*MdcAPX*, *MdcGR*, *MdMDHAR*, *MdDHAR1*, and *MdDHAR2*) were up-regulated (Fig. 4). Studies have shown that dopamine can alleviate plant stress response through modulation of physio-biochemical attributes and antioxidant defense systems, which is consistent with the results of this study [39, 40]. Hence, it is assumed that by declining oxidative damage, exogenous dopamine improved the chlorophyll synthesis and photosynthetic activity of plants under Cd stress and restored the growth of plants. In addition, stress conditions can increase the endogenous dopamine content of plants, and the application of exogenous dopamine can further increase endogenous dopamine content, which is the key to dopamine-driven resistance to abiotic stress [27, 28, 30, 40]. This study suggests that dopamine confers tolerance and that plants benefit from dopamine application under Cd stress.

When Cd is present in the cultivated environment, plant roots absorb Cd and transport it upward to aboveground parts, resulting in Cd enrichment of aboveground plant parts [41]. The root system is the essential organ of Cd uptake [8]. Cd accumulation in the aboveground part of the plant is related to Cd accumulation in plant roots [9]. Cd in the roots at much higher levels than in the leaves (Fig. 3). When Cd enters plant cells, it induces the production of ROS, and even cell death [35]. At the same time, plant roots can trap Cd, which weakens their capacity to transport Cd to the aboveground parts [35]. These processes affect Cd transport to the leaves. When dopamine was administered to apples, antioxidant enzymes activity was boosted, resulting in increased ROS scavenging potential and reduced Cd content. *MdNRAMP3*, *MdHMA4*, *MdFRO2-like*, and *MdHA7* are involved in the absorption and transport of Cd [8]. Higher expression levels of Cd detoxification gene helped to reduce Cd stress [8]. The expression level of Cd absorption genes was obviously decreased in DST plants, while the expression level of Cd detoxification genes was obviously increased (Fig. 4).

Exploration of the rhizosphere microbial community helped clarify the manner in which dopamine relieves Cd stress. The sequencing results showed that exogenous dopamine significantly affected the diversity and composition of microbial communities. We observed that Simpson index increased after exogenous dopamine treatment (Fig. 5). This high α-diversity demonstrates some level of resilience to environmental disturbance [42], allowing microbial community activity to be maintained [43]. Based on analysis of the assembly process of the sampled microbial community, we observed that exogenous dopamine mainly affected the assembly of the bacterial community under Cd stress (|βNTI| < 2). A previous study observed βNTI values between −2 and 2 for healthy rhizosphere soil, and the microbial community of healthy soil samples was assembled through stochastic processes [44]. Four basic processes can be used to show the microbial assembly processes occurring in different environments [45]. In this study, we used this framework to explore the effect of dopamine on the assembly process of the microbial community under Cd stress. Previous worked found that homogeneous selection dominated samples affected by heavy metals [44], while homogenizing dispersal accounted for a larger proportion of healthy soil samples [42]. Network analysis enables better evaluation of the interactions and identifies keystone species that make the largest impacts on microbial communities [46]. Our results showed much greater numbers of nodes and links in the bacterial network than the fungal network. Research has shown that soil bacteria are more active in response to various stress [47]. Microorganisms help their hosts maintain homeostasis and resist abiotic stress through the establishment of dense interaction networks [48]. The more complex the microbial community, the more resistant it is to environmental stress [49, 50]. The networks among bacteria and between bacteria and fungi became more complex after exogenous dopamine was applied, as demonstrated by increased numbers of nodes and links.

Exogenous dopamine can improve abiotic stress resistance by altering the rhizosphere microbial community and recruiting beneficial microorganisms [28]. Specific bacterial and fungal groups were obviously enriched to various extents in the treatments of this study. Based on Pearson correlation analysis, potentially beneficial microorganisms that were obviously enriched in treatment DST and were also obviously correlated with RGR and whole-plant Cd accumulation were selected (Fig. 8). Bacteria have the function of environmental bioremediation [51], and strengthening mutualistic symbioses among bacteria is particularly important to remediating heavy metal pollution [52]. One possible reason that exogenous dopamine alleviates Cd stress is that some taxa have the capacity to adsorb and detoxify Cd pollution, while others have the capacity to promote plant development. *Pseudoxanthomonas* is a common heterotrophic denitrifying bacterium belonging to the Proteobacteria [53]. Proteobacteria can remove heavy metals, and *Pseudoxanthomonas* shows effective resistance to chromium (VI) toxicity [54]. In addition, *Pseudoxanthomonas* has been applied to the removal of the organic pollutants polycyclic aromatic hydrocarbons [55]. *Aeromicrobium* has been demonstrated to degrade phenanthrene (PHE) in petroleum-contaminated soils, showing potential for use in soil remediation [56]. The increase in the abundance of *Bradyrhizobium* significantly increased nitrogen accumulation and the yield of maize [57]. *Frankia* is the main source of nodular symbiotic microorganisms [58]. As an Actinomycete, *Frankia* not only promotes plant growth but also has functions of heavy metal remediation and environmental protection [59]. LEfSe analysis indicated significant enrichment of *Saccharimonadales* in Cd-contaminated soils after the application of polymer amendments, which reduced Cd stress to plants [60]. The enrichment of *Novosphingobium* in clover roots can support complete rhizosphere degradation of PHE [61]. In addition, *Novosphingobium* has potential functions in disease resistance, soil repair and promotion of plant growth [62, 63]. *Streptomycetes* are widely distributed across diverse habitats and have important ecological functions [64], including persistent growth-promoting, antibacterial, and restorative functions [65].

A variety of beneficial interactions between the root system and the rhizosphere contribute greatly to plant growth [54]. Soil is a storage place for microorganisms as well as a repository of metabolites secreted by plant roots [48, 66]. These metabolites act as a bridge and play important roles in the interactions between plants and their environment [48, 66]. Different metabolites have different regulatory effects on rhizosphere microbial communities [67]. Soil metabolites provide carbon sources and signaling molecules that can stimulate rhizosphere microorganisms, which in turn help plants resist various stresses and promote plant growth [50]. The present study revealed dopamine-regulated metabolites associated with RGR (positive correlations) and Cd accumulation (negative correlations) (Fig. 12). These metabolites may function as drivers of the rhizosphere bacterial community. The application of exogenous dopamine may alleviate Cd stress by supporting the release of these metabolites, leading to enrichment of potentially beneficial microorganisms. In this study, based on analysis of the co-occurrence network between the relative abundances of differential metabolites and potentially beneficial microorganisms, several metabolites were found to have significant correlations with potentially beneficial microorganisms, suggesting that the recruitment of those microorganisms may be mediated by the differential metabolites. The interactions between bacteria and metabolites are an important factor in alleviating heavy metal toxicity in plants [68].

## Conclusion

**Figure 13:**
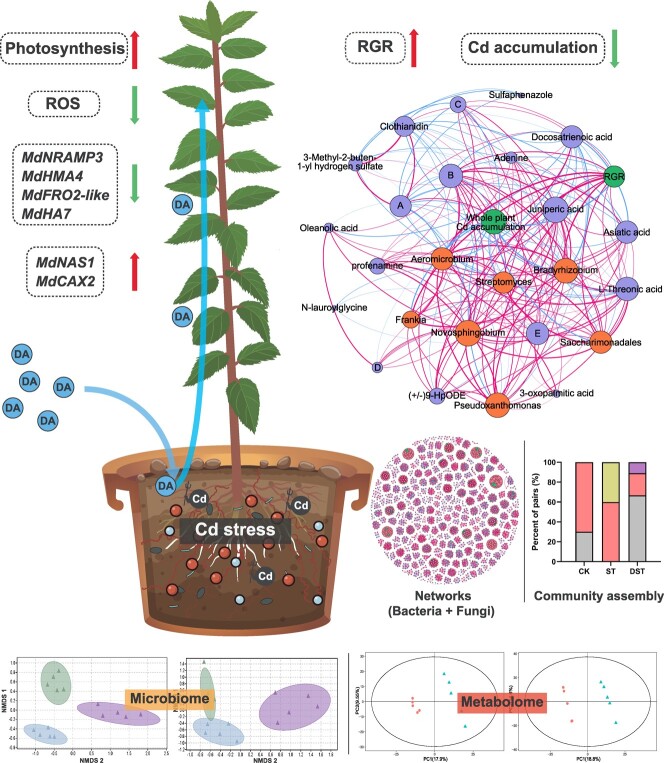
Diagram of the mechanism by which dopamine (DA) enhances cadmium stress tolerance in apple. Cd stress was induced by adding CdCl_2_ solution. DA reduced the level of ROS and enhanced photosynthesis under Cd stress. DA could reduce the Cd content by regulating the expression of related genes. In addition, DA could regulate rhizosphere microbial community and soil metabolic profile under Cd stress. Dopamine exerts positive (green arrows) and negative (red arrows) effects.

This study verified that exogenous dopamine could enhance the photosynthesis and activate the ROS scavenging system, thereby alleviating Cd stress in apple trees. Meanwhile, exogenous dopamine application inhibited the expression of Cd absorption genes, promoted the expression of Cd detoxification genes and reduced Cd accumulation in apple plants. Importantly, exogenous dopamine application significantly altered soil metabolites, as well as the diversity and composition of rhizosphere microorganisms associated with apple plants. Some potentially beneficial microorganisms and metabolites were recruited, which showed positive correlations with RGR or significant negative correlations with Cd accumulation. Tighter networks among rhizosphere microorganisms and changes in microbial community assembly processes were also observed when dopamine was applied to rhizosphere soil. Correlation analysis between the relative abundances of potentially beneficial microorganisms and soil differential metabolites indicated that dopamine-induced recruitment of potentially beneficial microorganisms may be driven by soil metabolite changes (Fig. 13). These findings provide the innovative strategies to mitigate the threat of Cd stress to apple production, and the dopamine-related beneficial microorganisms will bring great benefits to the study of plant resistance. However, the actual application effect of dopamine in the field experiments needs to be further studied.

## Materials and methods

### Experimental treatments

One-year-old healthy apple seedlings (*M. hupehensis*) with heights of approximately 50 cm were used as the experimental material. Healthy seedlings of the same size were selected for 30 days of concentration screening. The dopamine concentrations tested included 0, 25, 50, 100, and 200 μM, and the concentration of Cd applied was 200 μmol/L CdCl_2_·5H_2_O. After 30 days, TD, PL, TCC, REL, production of O_2_^•−^, T-AOC, and H_2_O_2_ content were determined.

Based on the results of screening, 100 μM of dopamine was used as the final experimental concentration (Figs. S4 and S5, see online supplementary material). Consistently healthy seedlings were selected. The seedlings were divided into three groups, each containing 40 seedlings, for a total of 120 seedlings: 0 μmol/L CdCl_2_·5 H_2_O (CK); Cd treatment with 200 μmol/L CdCl_2_·5H_2_O (ST); and Cd treatment with 200 μmol/L CdCl_2_·5H_2_O + 100 μmol/L dopamine (DST). Seedlings were treated every 5 days for 60 days.

### Measurement of plant growth parameters

The PL and TD in each treatment were measured every 15 days during the experiment. At day 60, 10 seedlings showing consistent growth from each treatment were carefully removed from their pots and divided into leaves, stems, and roots, and RGR and TDW were calculated [69].

### Measurement of leaf physiological indexes and dopamine content

Healthy mature leaves were selected every 15 days during the experiment, and TCC and REL were determined [28]. At day 60, fresh young leaves of the same size were harvested for leaf histochemical staining according to the kit instructions (Servicebio, Wuhan, China). The NPQ, Fv/Fm, H_2_O_2_, O_2_^•−^, T-AOC, APX, POD, CAT, and SOD were determined according to the methods of Cao *et al.* [28]. The concentration of dopamine was analysed using high-performance liquid chromatography (HPLC; LC-2010, Shimazu, Japan) [28].

### Quantification of gas exchange

The Tr, Pn, Gs, Ci were monitored with a Li-Cor portable photosynthesis system on sunny days. Total 30 leaves were collected for measurement [69].

### Determination of cd content

At day 60, samples were exposed to staining solution (acetone, diphenylthiourea, glacial acetic acid) for 2 h. Then, Cd was observed with an optical microscope (BX63, Olympus, Japan) [40]. Cd concentration was determined as described by Huang *et al.* [70].

### qRT-PCR analysis

qRT-PCR was conducted using Fast SuperEvaGreen qPCR Master Mix (S2008S) (US Everbright Inc, SuZhou, China) following the method of Cao *et al.* (2022) [28]. The primer sequences are presented in Table S1 (see online supplementary material).

### Microbial community analysis

Loose soil was shaken off. Approximately 30 g of the root system with soil was placed into sterilized NaCl solution. Five plants were mixed into a sample, with five replicates analysed per treatment. Specific primers were used for PCR amplification after total DNA extraction. The 16S rRNA primer sequences were 338F (5′- ACTCCTACGGGAGGCAGCA −3′) and 806R (5′- GGACTACHVGGGTWTCTAAT −3′). The internal transcribed spacer (ITS) primer sequences were ITS5 (5′- GGAAGTAAAAGTCGTAACAAGG −3′) and ITS2 (5′- GCTGCGTTCTTCATCGATGC -3′). After amplification, the PCR products were quantified, and then the library construction was constructed. QIIME2 version 2019.4 and the official tutorial were used to modify and improve the process of biological information analysis for microbial groups. The DADA2 plug-in was used for quality filtering, de-noising, concatenation, and chimera removal. The obtained sequences were merged, and characteristic sequence amplicon sequence variants (ASVs) and abundance data tables were generated. The raw sequencing data were submitted to the NCBI Sequence Read Archive (SRA) database under the accession number SRP425616.

### Soil metabolome detection

Samples were taken after gently shaking the roots, and all rhizosphere soils within 5 mm of the root surface were collected. The roots of each group of five plants were mixed into a sample with five replicates per treatment. Analyses were performed by Personal Biotechnology Co. Ltd (Shanghai, China). The raw MS data (wiff.scan files) were converted to MzXML files and processed. SIMCA-P 14.1 (Umetrics, Umea, Sweden) was used for PLS-DA [71].

### Bioinformatics and statistical analysis

Simpson index values were calculated using QIIME2. The LEfSe was used to determine the types of microorganisms enriched [72]. Then, the βNTI between pairs of samples was calculated, and the phylogenetic pattern of the rhizosphere microbiome was evaluated. Sample pairs with |βNTI| > 2 are expected to result from deterministic processes, while pairs with |βNTI| < 2 are likely governed by stochastic processes. The calculation method was described by Zhang *et al.* (2022) [72]. A microbial inter-domain network was constructed using filtered ASVs. The correlations between ASVs were determined using R version 3.6.0. Co-occurrence networks were visualized and the number of nodes and links were calculated using Gephi 9.2 [46].

Differences were determined using SPSS 26.0 (IBM Corp., Armonk, NY, USA) software. The graphs were constructed using GraphPad Prism 9.0 (San Diego, CA, USA). Tukey’s multiple range tests were used to check the significant difference between means (*P* < 0.05).

## Acknowledgments

This work was supported by the National Natural Science Foundation of China (No. 31901964), the Science and Technology Project of Hebei Education Department (No. BJK2022012), and the Earmarked fund for the China Agricultural Research System (No. CARS-27).

## Author contributions

B.L., Y.C., and P.D. conceived and designed the experiments. Y.C. and P.D. performed the experiments with assistance from J.Z. and J.J. Y.C. and P.D. analysed the data and drafted the manuscript. J.X. provided laboratory apparatus. B.L. provided financial support and revised the manuscript. All authors read and approved this manuscript.

## Data availability

Data will be made available on request.

## Conflict of interest statement

The authors declare that they have no competing interests.

## Supplementary data

Supplementary data is available at *Horticulture Research* online.

## Supplementary Material

Web_Material_uhad112
