# Supplementary material for: Dopamine alleviates cadmium stress in apple trees by recruiting beneficial microorganisms to enhance the physiological resilience revealed by high-throughput sequencing and soil metabolomics
Source: Hortic Res. 2023 May 22;10(7):uhad112. doi: 10.1093/hr/uhad112 (PMC10419553; doi:10.1093/hr/uhad112)
Supplement: Web_Material_uhad112 [file web_material_uhad112.zip › R1-Supplymental material.docx]

**Coupling high-throughput sequencing and soil metabolomics to investigate the mechanisms through which dopamine enhances cadmium tolerance in apple**

**Running title:** Dopamine enhances apple cadmium resistance

Yang Cao^&^, Peihua Du^&^, Jiran Zhang, Jiahao Ji, Jizhong Xu, Bowen Liang*

College of Horticulture, Hebei Agricultural University, Baoding, Hebei 071001, China

***Corresponding author**:

E-mail address: lbwnwsuaf@126.com or lbw@hebau.edu.cn (B. L.).

^&^ These authors contributed equally to this work.

The e-mail addresses of the contributing authors

Yang Cao: caoyang126129@163.com (Y. C.)

Peihua Du: dupeihua1997@163.com (P. D.)

Jiran Zhang: xiaoyou2xx@163.com (J. Z.)

Jiahao Ji: ihaosymphony@gmail.com (J. J.)

Jizhong Xu: yyxjz@hebau.edu.cn (J. X.)

**Table S1** **The primes used for qRT-PCR**

| Gene | Forward primer | Reverse primer |
| --- | --- | --- |
| *MdSOD* | GGGAGATGGCCCAACTACTG | TTGCCAAGGTCATCAGGGTC |
| *MdPOD* | CCAACAAATGTGTCCCAAAAATG | CCTGGTCCGAGGTAAATAATCC |
| *MdCAT* | TGAAACCAAATCCAAAGACCA | TTCCATGTGCCTGTAGTTGAGTG |
| *MdAPX* | AACTACAAGGGATGAAGCC | CAACGAGGATGATAACCAG |
| *MdDHAR 1* | AGTGGACGGTTCCAGCAGA | TTCCCATCCCGCAATCAC |
| *MdDHAR 2* | CCACCATCAAACACCACCTT | TTGGGAACAGTAACGGAAGC |
| *MdcGR* | GTTCAGCGACAAGGCGTAT | TCAACCGATTTCCATTTCC |
| *MdMDHAR* | CCATACTTCTATTCCCGCTCCT | CGACCACCTTCCCGTCTTT |
| *MdNRMP3* | GACGACGACTGCCAAACGCCG | CCACAGCACCATCCTCGCCCA |
| *MdHMAA4* | GGCATCCGCTCGTTT | TTTGGGGTTTACAGGCT |
| *MdFRO2-like* | GGGGTTAATCTTCTGCTCTTCTCAT | CAGAGGAGCCTTCACAACCACT |
| *MdHA7* | TGGCAGGGATGGATGTTC | TGCAGTTCTTTTATCGGTAGG |
| *MdNAS1* | CGATGTTTCCAAGTTATGCCAA | TTCTCCAAAAGTCCTTCTGCCT |
| *MdCAX2* | CAATTTATGCACTGAAACGTGG | CTGAAGAATGCACAACCAAGC |
| *MdMDH* | CGTGATTGGGTACTTGGAAC | TGGCAAGTGACTGGGAATGA |


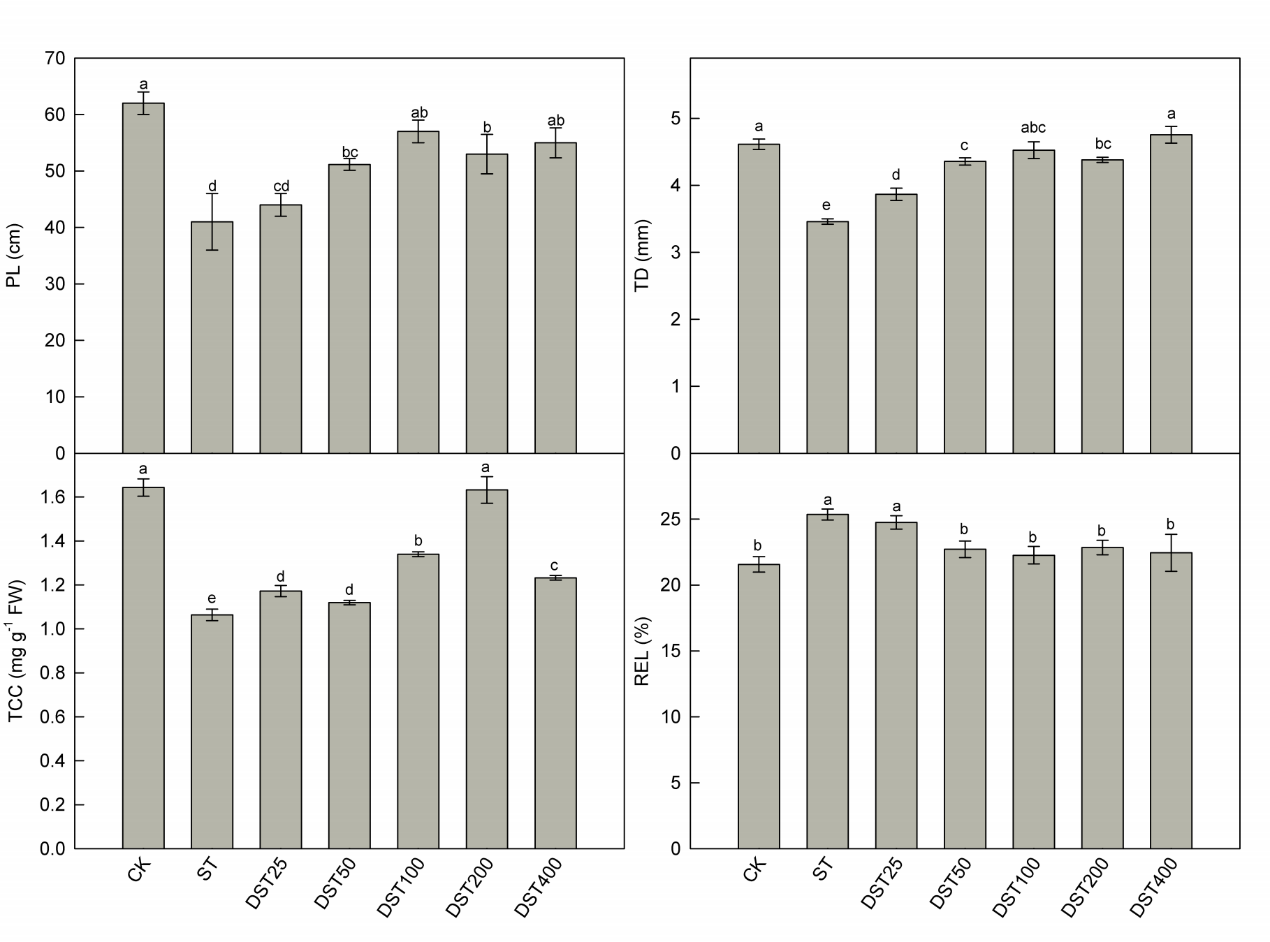


**Figure S1.** **Effects of different concentrations of dopamine on plants growth, TCC and REL under Cd stress.** DST25, Cd stress with 25 μM dopamine; DST50, Cd stress with 50 μM dopamine; DST100, Cd stress with 100 μM dopamine; DST200, Cd stress with 200 μM dopamine; DST400, Cd stress with 400 μM dopamine.


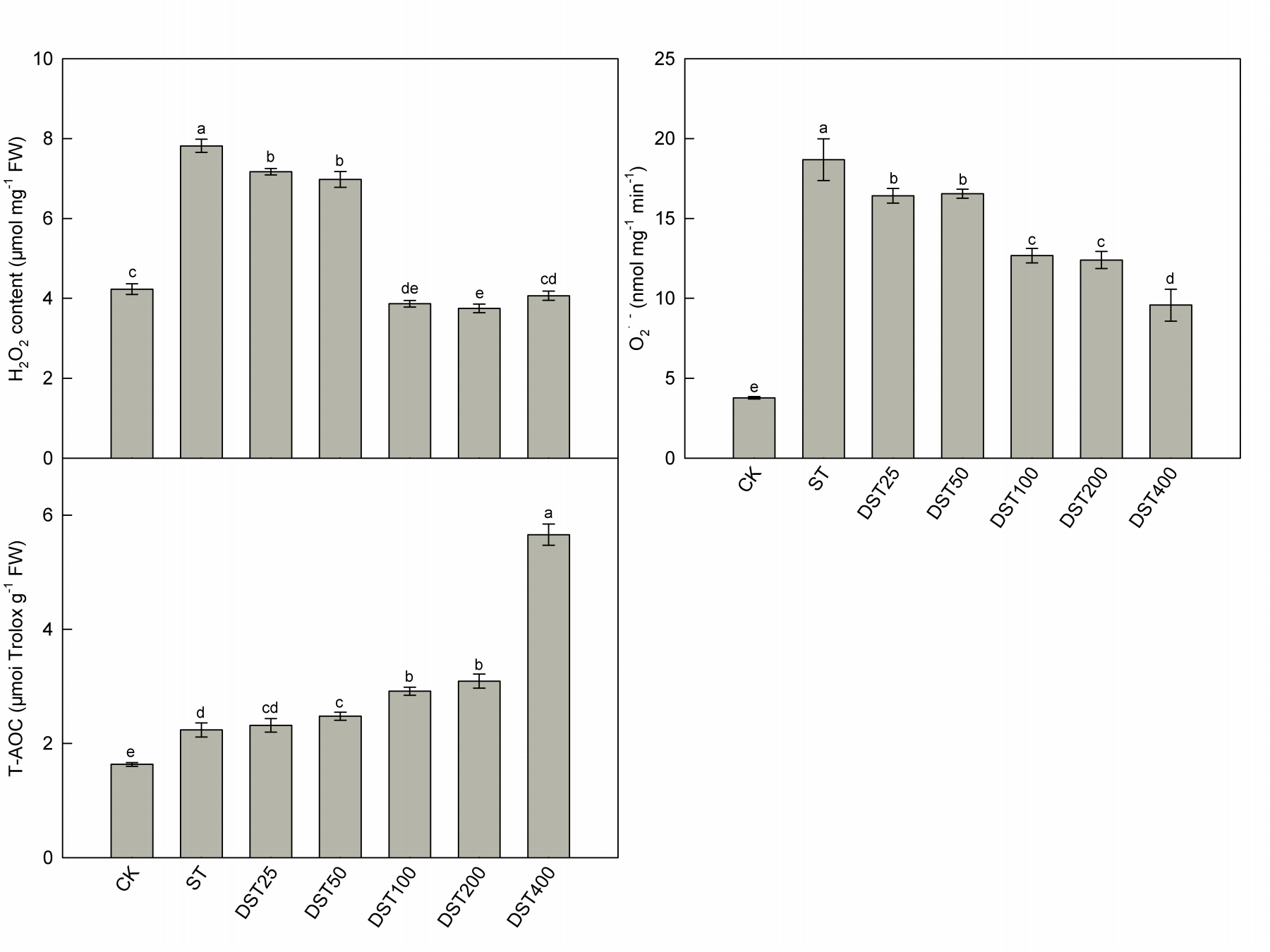


**Figure S2**. **Effects of different concentrations of dopamine on H_2_O_2_ content, the O_2_^.-^ production rate and T-AOC under Cd stress.** DST25, Cd stress with 25 μM dopamine; DST50, Cd stress with 50 μM dopamine; DST100, Cd stress with 100 μM dopamine; DST200, Cd stress with 200 μM dopamine; DST400, Cd stress with 400 μM dopamine.


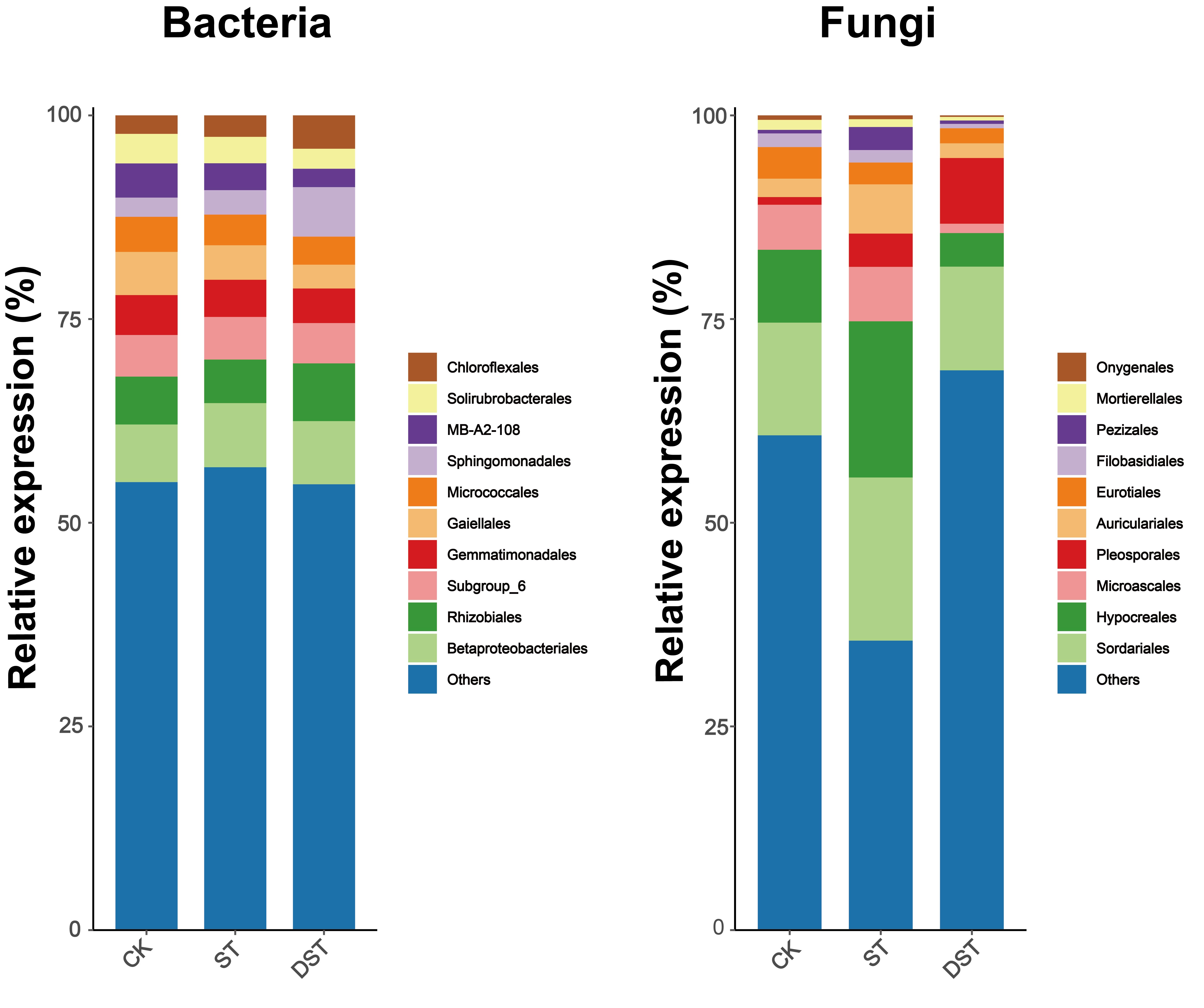


**Figure S3. Relative abundances of different bacterial and fungal taxa at the order level.** CK, control; ST, Cd stress; DST, Cd stress with 100 μM dopamine.


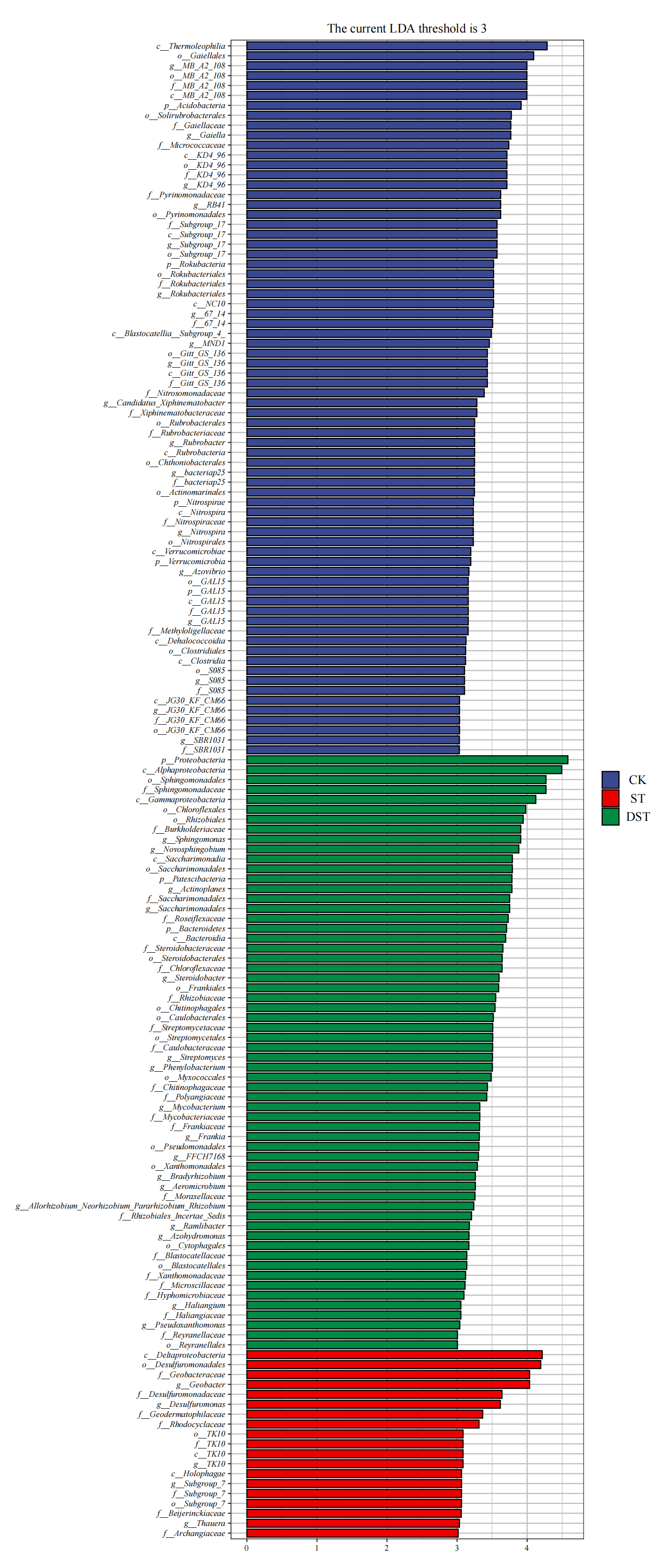


**Figure S4. Indicator** **bacteria (LDA > 3).** p: phylum, o: order, c: class, f: family, g: genus. CK, control; ST, Cd stress; DST, Cd stress with 100 μM dopamine.


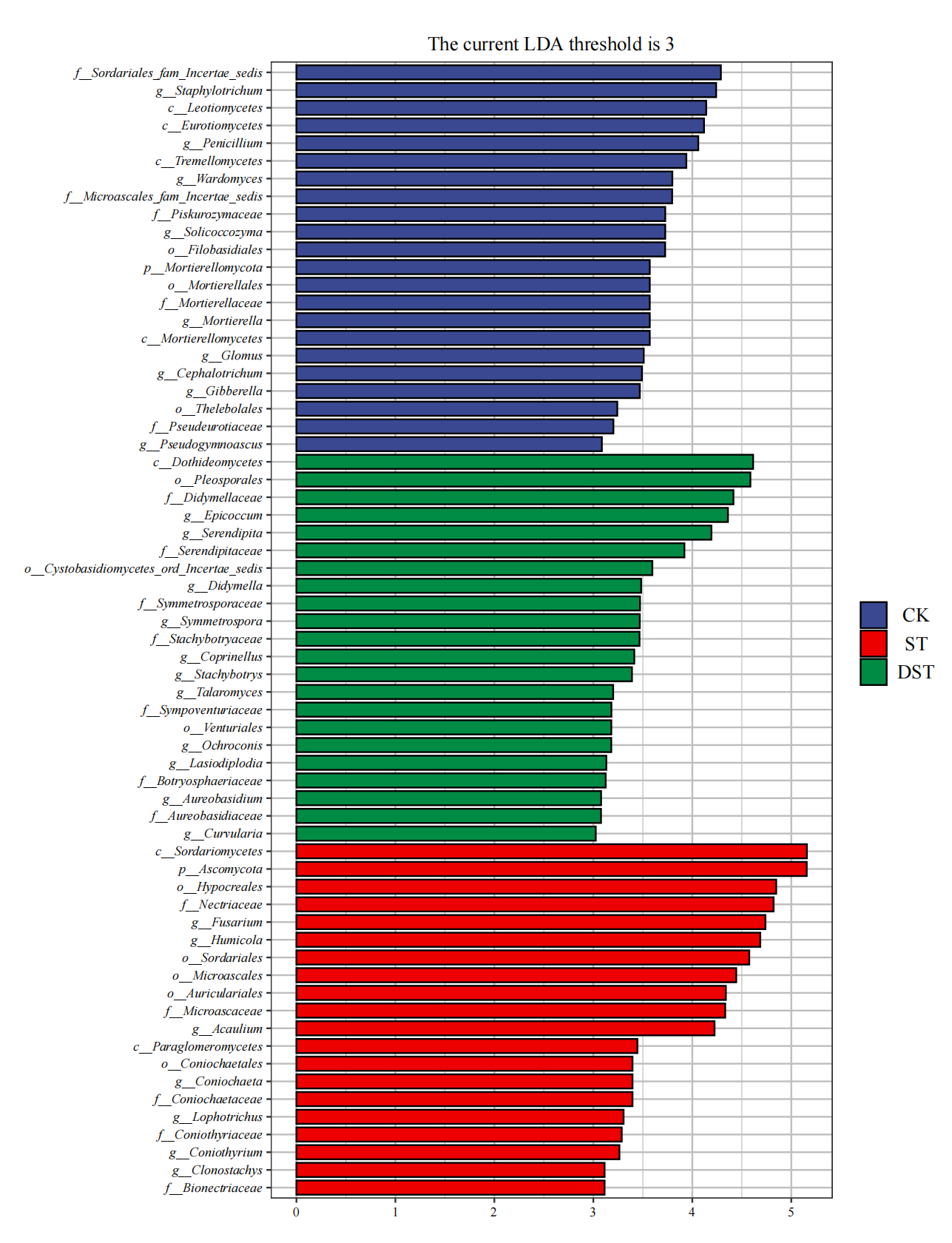


**Figure S5.** **Indicator fungi (LDA > 3).** p: phylum, o: order, c: class, f: family, g: genus. CK, control; ST, Cd stress; DST, Cd stress with 100 μM dopamine.
